# Surveillance of antibiotic resistance among uropathogens in Aljouf region northern Saudi Arabia

**Published:** 2019-12

**Authors:** Ibrahim Taher, Abdulrahman Almaeen, Hassan Aljourfi, Eyad Bohassan, Ahmed Helmy, Eman El-Masry, Baraka Saleh, Nawaf Aljaber

**Affiliations:** 1 Department of Pathology, College of Medicine, Jouf University, Sakaka, Saudi Arabia; 2 Department of Medicine,College of Medicine, Jouf University, Sakaka, Saudi Arabia; 3 Department of Tropical Medicine & Gastroenterology, Assiut University Hospitals, Assiut, Egypt; 4 Department of Microbiology, Microbiology Laboratory, Prince Mutaib Hospital, Sakaka, Saudi Arabia

**Keywords:** Urinary tract infection, Gram-negative bacteria, Gram-positive bacteria, Antimicrobial susceptibility

## Abstract

**Background and Objectives:**

Urinary tract infections are common health problem affecting millions worldwide. Antibiotic resistance among uropathogens (Ups) is prevalent in many countries. In the absence of any available data in the region, this hospital-based study investigated the pattern, frequency and susceptibility of Ups at Prince Mutaib Bin Abdulaziz Hospital, Aljouf Region, Saudi Arabia.

**Materials and Methods:**

A retrospective assessment of UPs and their antibiotics susceptibility was conducted from January 2017 to December 2017 using the fully automated Vitek2 system (BioMérieux, France).

**Results:**

Among the 415 uropathogens isolates, the most prevalent bacteria were Gram-negatives comprising 137 (51%) *E. coli*; 46 (17.2%) *Klebsiella* spp.; 30 (11.2%) *Pseudomonas* spp.; 25 (9.3%) *Proteus* spp.; 14 (5.2%) *Acinetobacter baumanii* and 16 (5.9%) others. On the other hand, *Enterococcus* spp. were predominant among Gram-positive isolates representing 54 (36.7%), 47 (32.0%) *Staphylococcus* spp., 22 (15.1%) *Streptococcus* spp., and 13 (8.8%) *S. aureus*, and 11 (7.5%) others. Gram-negative Ups showed multidrug resistance towards the majority of the tested antimicrobials (ampicillins, cephalosporins, fluoroquinolones, trimethoprim-sulfamethoxazole, fosfomycin, aztreonam, and nitrofurantoin). While high resistance patterns by Gram-positives was also seen against cephalosporins, penicillins, amoxicillin-clavulanic acid, trimethoprim-sulfamethoxazole, clindamycin, erythromycin and tetracycline.

**Conclusion:**

The observed widespread multidrug resistance clearly warrant implementing stricter control measures, local guidelines of antimicrobials usage, and continuous epidemiological surveys at hospitals and communities.

## INTRODUCTION

Urinary tract infections (UTIs) are very common wide-spread affecting about 150 million people yearly (1). Gram-negative bacteria cause most UTIs, with *E. coli* being the most commonly encountered followed by other Gram negatives (e.g. *Proteus, Klebsiella, Pseudomonas, Enterococcus*), and Gram-positives such as: Group B Streptococci, and *Staphylococcus saprophyticus* (2, 3). A wide range of antibiotics resistance patterns have been reported in different parts of the world, and the empirical use of antimicrobials has proved to be an important predictor of resistance against the antimicrobial drugs (AMDs) used (4). The increased incidence of UTIs and the need by physicians to start patients’ care before carrying out any microbiological investigations had often led to empirical use of broad-spectrum antibiotics in most communities worldwide (5). This has widely been practiced despite the fact that urine specimens can easily be obtained, and urine culture is a relatively straightforward technique.

The easy access to affordable AMDs results in rapid selection of antimicrobial drug resistance (AMR), which is a difficult one to strike (6). Therefore, a continuous surveillance of the usage of these drugs is always required. In return, this can guide decision makers; AMDs stewardship programs; guidelines for empirical treatment that allow the monitoring of trends in AMR and the potential impact of interventions in reducing its development (7). Unfortunately, this may prove to be difficult to achieve particularly in low income developing countries, due to limited financial and human resources and the poor quality of microbiology laboratories (8).

AMR amongst UPs in different geographical regions of the world is recognized as a serious global health problem (9, 10). Regional surveillance studies to accurately identify UPs and determining their resistance to antibiotics are of paramount importance for the efficient management of patients, leading to clinical and financial benefits such as reducing mortality rates and hospitalization costs (11).

Earlier studies have shown increased resistance patterns among UPs against commonly used AMDs in other regions of Saudi Arabia (12–15). The most commonly encountered UPs were *E. coli, Klebsiella* spp., *Pseudomonas* spp., and others. Multiple AMR has widely been reported against ampicillin, trimethoprim-sulfamethoxazole, cephalosporins, gentamicin, and ciprofloxacin (12–14). ESBLs production by *E. coli* and *K. pneumoniae* in addition to other risk factors, such as diabetes, recurrent UTI, previous use of antibiotics, previous hospitalization, underlying renal disease and renal transplantation, have all been significantly associated with the wide-spread of antibiotic resistance amongst such pathogens (13, 14).

Although there are available data concerning AMR in other regions in Saudi Arabia, no study was conducted at Aljouf region. Therefore, the present hospital-based study aimed to investigate the pattern, frequency and susceptibility of UPs at Prince Mutaib Hospital at Aljouf Region in northern Saudi Arabia.

## MATERIAlS AND METHODS

### Study design.

A retrospective hospital-based study of UPs and their susceptibility patterns to antibiotics were carried out during January 2017 to December 2017 at Prince Mutaib Hospital, Sakaka, Aljouf, Saudi Arabia. The study included 415 patients of whom 245 (59.0%) were females and 170 (41.0%) males; 376 (90.6%) were Saudis and the other 39 (9.4%) were non-Saudis. The mean ± SD age of patients was 47.0 ± 24.4 years (range: 55 days to 100 years).

### Ethical approval.

The work has been carried out in accordance with The Code of Ethics of the World Medical Association (Declaration of Helsinki, amended version 2013). An informed consent was obtained from all subjects, and their privacy rights were observed. The study was approved by the Research Ethics and Advisory Committees of the College of Medicine, Jouf University.

### Sampling procedure.

During the study period all urine samples were collected in boric acid containers and sent to the Microbiology Laboratory of Prince Mutaib Hospital. The indication of urine analysis was suspected or having UTIs, presence of urinary symptoms, as well as urine cultures taken preoperatively from asymptomatic patients not having UTIs whether they are inpatients or outpatients.

### Identification and determination of antimicrobial susceptibility.

All the urine specimens were cultured on Cystine Lactose Electrolyte Deficient agar (CLED) plates (Oxoid, Basingstoke, UK) and incubated for 24–48 hours at 37°C. All the isolates were identified and tested for their antibiotic sensitivity profiles using an automated VITEK-2 Machine (BioMérieux, Marcy-I’Étoile, France) which was the only available system in the hospital. The Vitek-2 is a fully automated system that depends on the microbial growth technology. The reagent cards used contain 64 wells with different test substrates that are used to measure metabolic activities e.g. enzyme hydrolysis, alkalinization, acidification, and growth in the presence of inhibitory substances. The bacterial suspensions were prepared from pure cultures using 0.5% sterile NaCl and cards were inoculated and incubated accordingly. All bacterial suspensions were prepared to be at a concentration of 0.5–0.63 McFarland using the VITEK DensiCHEK colorimeter (BioMérieux).

The AST-GN72 and AST-GP71 cards were used for both Gram-negative and Gram-positive bacteria respectively. Results of AMR were interpreted according to the recommendation of the Clinical and Laboratory Standards Institute (19). The VITEK-2 system manufacturer’s guidelines were followed in order to determine both the extended-spectrum-β-lactamase (ESBL) and the methicillin-resistant *Staphylococcus aureus* (MRSA) activities. For quality control purposes *E. coli* ATCC 25922 and *S. aureus* ATCC 29213 were used.

### Statistical analyses.

Data were initially collected in a pre-formed Data Collection Form prior to being entered in Microsoft Excel Spreadsheet. A descriptive and analytical statistical analysis was performed using the Statistical Package for Social Sciences (SPSS Inc., Chicago, IL, USA) version 22. A p value <0.05 was considered statistically significant. Discrete variables were expressed as frequencies and percentages or mean ± standard deviation (SD) as appropriate. Comparisons between groups were done using Chi-Square or Fisher’s exact test or Mann Whitney-*U* test as appropriate.

## RESUlTS

Of the total urine samples processed over a period of one year; 1640 (76.7%) yielded significant microbial growth of which 960 (58.5%) were Gram-negative bacteria, 671 (40.9%) Gram-positive, and only 9 (0.5%) were *Candida* spp. However, we are presenting the results of 415 samples which we have managed to retrieve. Out of these 147 (35.4%) and 268 (64.6%) were Gram-positive and Gram-negatives respectively.

Inpatients and outpatients represented 228 (54.9%) and 187 (45.1%) respectively (Table 1). In comparison, patients infected with Gram-negative isolates were found to be significantly older than those with Gram-positive (50.2 ± 25.7 versus 41.1 ± 20.5; p = 0.001) regardless of being inpatient or outpatient (p = 0.000, Table 1). However, there was no significant differences between patients with either isolates regarding gender and nationality, p = 0.233 and p = 0.703 respectively (Table 1). Diagnoses on admission included a wide spectrum of diagnoses that are encountered in both the outpatient and inpatient Departments of a secondary healthcare hospital including chronic renal, metabolic, chest, heart, neurological disorders, and acute illnesses, with suspected UTIs.

**Table 1.** Demographic characteristics of patients

**Variable**	**Unit/Category**	**Patients with Bacterial Isolates**			P value^*^

		**All (n=415)**	**Gram −ve (n=268)**	**Gram +ve (n=147)**	
Age	Years	46.8 ± 24.5	50.2 ± 25.7	41.1 ± 20.5	0.001
Gender	Male	170 (41.0)	117 (43.7)	53 (36.1)	0.233
	Female	245 (59.0)	151 (56.3)	94 (63.9)	
Nationality	Saudi	376 (90.4)	244 (91.0)	132 (89.8)	0.703
	Non Saudi	39 (09.6)	24 (09.0)	15 (10.2)	
Setting	Inpatient	228 (66.7)	100 (37.3)	87 (59.2)	0.000
	Outpatient	187 (33.3)	168 (62.7)	60 (40.8)	

Data are expressed as mean SD or n (%) as appropriate.

* Gram −ve versus Gram +ve.

These isolates were distributed as follows: 137 (51.1%) *E. coli*; 46 (17.2%) *Klebsiella* spp.; 30 (11.2%) *Pseudomonas* spp.; 25 (9.3%) *Proteus* spp.; 14 (5.2%) *Acinetobacter baumannii*, and 16 (6.0%) other Gram-negative organisms (Table 2). In comparison, the Gram-positive isolates were 54 (36.7%) *Enterococcus* spp.; 47 (32.0%) Coagulase-negative *Staphylococci* (CNS); 22 (15.1%) *Streptococcus* spp., 13(8.8%) *S. aureus*; and 11 (7.5%) other Gram positive organisms (Table 2).

**Table 2.** Frequency and percentages of the different isolated uropathogens (n=415)

**Gram stain**	**Organism**	**Frequency**
Gram-negative	All	268/415 (64.6)
	*E. coli*	137 (51.1) #
	*Klebsiella* spp.	46 (17.2) #
	*Proteus* spp.	25 (9.3) #
	*Pseudomonas* spp.	30 (11.2) #
	*Acinetobacter baumanii*	14 (5.2) #
	Others	16 (6.0) #

Gram-positive	All	147/415 (35.4)
	*Staphylococcus* spp.	47 (32.0) ^*^
	*Staphylococcus aureus*	13 (8.8) ^*^
	*Enterococcus* spp.	54 (36.7) ^*^
	*Streptococcus* spp.	22 (15.1) ^*^
	Others	11 (7.5) ^*^

Data are expressed as n (%).

# = % from all Gram negative isolates.

* = % from all Gram positive isolates.

**Fig. 1.**
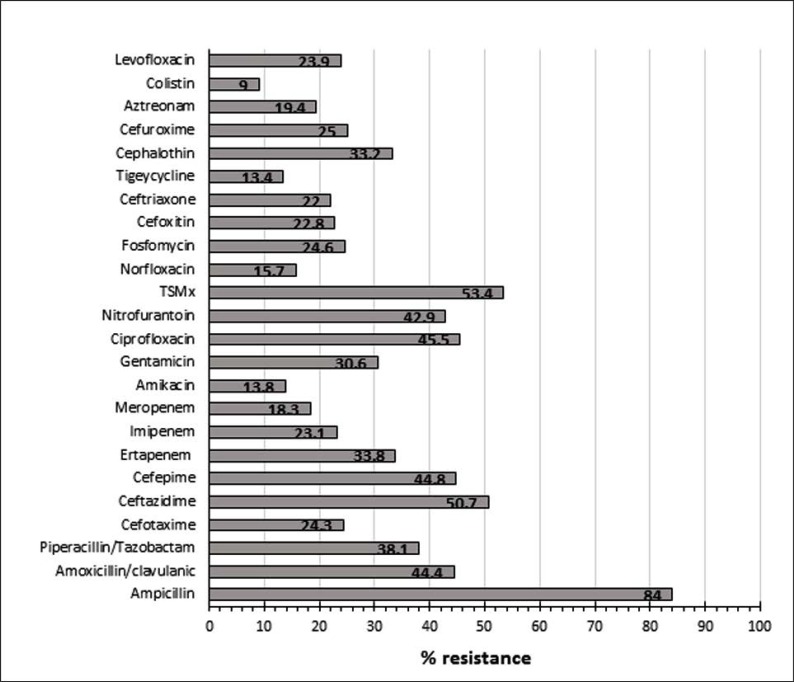
Percentage resistance pattern of Gram-negative isolates to the studies antimicrobial drugs. TSMx = Trimethoprim-sulphamethoxazole.

Table 3 and Fig. 1 summarized the AMDs susceptibility patterns of all Gram-negative isolates. It shows a high resistance rate against ampicillin (84.0%), trimethoprim-sulphamethoxazole (53.4%), (50.7%), ciprofloxacin (45.5%) and nitrofurantoin (42.9%). Lower rates were recorded towards colistin, amikacin, norfloxacin, meropenem and ceftriaxone with an overall resistance rate of 9.0%; 18.513.8%, 15.7%, 18.3% and 22.0% respectively. Of the 137 *E. coli* isolates, 57 (41.6%) were designated as extended spectrum β-lactamase (ESBL) producers compared with 8 out 46 (17.4%) among *Klebsiella* spp., 3 out of 25 (12.0%) *Proteus* spp., and none of the *Pseudomonas* spp., and *Acinetobacter* spp., (p<0.001) as confirmed by the Vitek 2 System.

**Table 3.** Percentage resistance pattern of Gram-negative uropathogens (n=136).

**Drug**	***E. coli***	***Klebsiella* spp.**	***Pseudomonas* spp.**	***Proteus* spp.**	***Acinetobacter* spp.**
Ampicillin	85.7	90.9	100	95.4	100
Amoxicillin/clavulanic	30.2	50.0	95.8	55.0	100
Piperacillin/Tazobactam	29.9	35.6	46.7	56.0	82.7
Cefotaxime	62.9	57.1	F	F	F
Ceftazidime	37.5	46.7	80.0	64.0	82.7
Cefepime	37.5	41.3	51.7	68.0	82.7
Ertapenem	31.5	41.0	86.4	47.1	100
Imipenem	16.3	13.6	50.0	24.0	85.7
Meropenem	07.4	23.9	43.3	12.0	82.7
Amikacin	9.50	10.9	20.0	12.0	82.7
Gentamicin	18.2	23.9	36.7	64.0	75.0
Ciprofloxacin	37.5	45.6	43.3	68.0	78.6
Nitrofurantoin	32.0	43.6	60.9	95.0	100
TSMx	40.0	46.7	96.6	80.0	57.1
Norfloxacin	23.2	46.7	72.7	100	F
Fosfomycin	58.1	35.3	90.1	66.7	F
Cefoxitin	27.4	31.0	100	33.3	100
Ceftriaxone	46.0	54.5	100	50.0	70.0
Tigecycline	13.3	5.60	90.0	100	F
Cephalothin	62.3.	59.1	100	72.3	100
Cefuroxime	45.5	56.5	100	F	100
Aztreonam	40.0	55.0	64.3	F	100
Colistin	F	08.3	44.4	100	0
Levofloxacin	44.4	44.0	68.4	77.8	80.0

F= very few to report. TSMx = Trimethoprim/sulfamethoxazole.

**Fig. 2.**
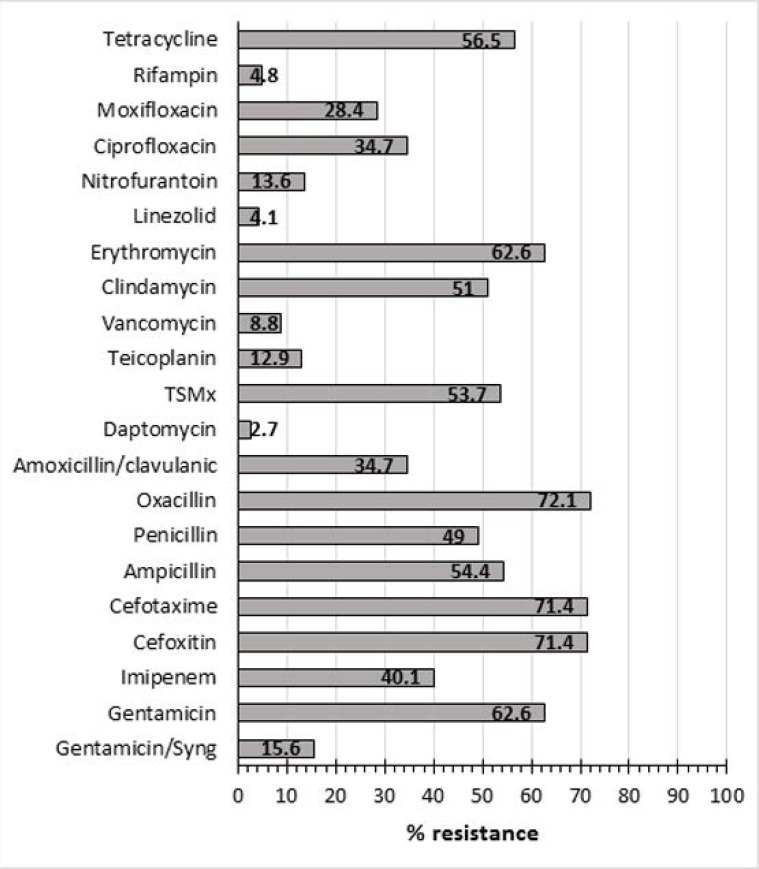
Percentage resistance pattern of Gram-positive isolates to the studies antimicrobial drugs. TSMx = Trimethoprim-sulphamethoxazole.

Table 4 and Fig. 2 show the drug resistance pattern of the Gram-positive bacteria against the AMDs tested. A high AMDs resistance pattern was observed towards oxacillin (72.1%), cefoxitin (71.4%), cefotaxime (71.4%), erythromycin (62.6), gentamicin (62.6%), tetracycline (56.5%), trimethoprim-sulphamethoxazole (53.7%) and clindamycin (51.0%). However, lower resistance rates by Gram-positive UPs were recorded for daptomycin (2.7%), linezolid (4.1%), vancomycin (8.8%) and rifampicin (4.8%). Only 2 of the 13 *S. aureus* isolates (15.4%) were marked as methicillin-resistant *S. aureus* (MRSA) and 16 out of 26 *S. haemolyticus* (61.5%) were found to be methicillin-resistant *S. haemolyticus* (MRSH)

**Table 4.** Percentage resistance pattern of Gram-positive uropathogens (n=136)

**Drug**	***Staphylococcus* spp.** **n=60**	***Streptococcus*** **spp.** **n=22**	***Enterococcus* spp.** **n=54**
Gentamicin/Syng	56.6	20.0	38.3
Gentamicin	0	71.4	90.6
Imipenem	78.9	33.3	30.8
Cefoxitin	97.6	90.9	100
Cefotaxime	78.4	83.3	100
Ampicillin	100	42.9	29.2
Penicillin	98.0	29.4	77.8
Oxacillin	80.0	83.3	100
Amoxicillin/clavulanic	69.8	F	71.4
Daptomycin	02.1	05.9	04.7
TSMx	35.6	66.7	90.2
Teicoplanin	11.9	12.5	16.7
Vancomycin	06.8	05.6	13.0
Clindamycin	96.6	40.0	92.9
Erythromycin	75.5	31.3	79.2
Linezolid	01.9	05.0	07.5
Nitrofurantoin	13.6	08.3	17.0
Ciprofloxacin	36.4	09.1	51.1
Moxifloxacin	09.4	33.3	32.0
Rifampin	07.5	66.7	F
Tetracycline	47.4	33.3	77.4

F= very few to report. TSMx = Trimethoprim/sulfamethoxazole.

## DISCUSSION

UTIs are mostly treated empirically, and the criterion for the selection of AMDs is most commonly based on the most likely pathogen and its expected AMR pattern in a given locality (16). In the present study, *E. coli* was the most frequently isolated UP among Gram-negative isolates followed by *Klebsiella* spp., *Pseudomonas spp*., and others. This is somewhat similar to the findings of many previous studies in that 75–90% of UTIs were due to *E. coli*; whereas, *Staphylococcus* spp., were the most common Gram-positive isolates (17–20).

Elderly women are known to be prone to develop asymptomatic bacteriuria and recurrent UTIs, which have been linked with risk factors such as diabetes among this age and gender category (18). Likewise, our result showed similar findings to other studies in that 59% of UPs encountered in this study were among female patients. This is probably due to women-related anatomical and physiological changes in addition to other likely risk factors (2, 21–23). In addition, due to the likely recurrent attacks of UTIs among this group leading to frequent use of AMDs, taking the wrong AMDs for asymptomatic bacteriuria, or treatment of others infections (24) could all play part in the occurrence of UTIs as well as the development of AMR. Similarly, previous hospitalization and history of previous intake of AMDs may well affect the pattern and the sensitivity profile of the UPs in these patients.

As the etiological agents of UTIs and their susceptibility/resistance patterns vary according to geographical locations (1, 24), there is a continuous need for periodic monitoring of the UPs and antibiotic resistance patterns. This will, consequently, give updated recommendation for the optimal empirical therapy of UTIs (25). Our Gram-negative UPs showed an overall AMR rates ranging from 9.0% for colistin to 84.0% for ampicillin. A high resistance rate was recorded against second, third and fourth generation cephalosporins ranging from 22.0% to 50.7%. A similar resistance pattern was also shown against the majority of the tested drugs including fluoroquinolones, fosfomycin, amoxicillin/clavulanic acid, trimethoprim/sulfamethoxazole, nitrofurantoin, and others. In general, over 40% of our G-negative UPs (*E. coli, K. pneumoniae, Pseudomonas* spp., *Proteus* spp., and *Acinetobacter* spp.) were resistant to cephalosporins. Similar studies in Saudi Arabia, have shown that bacterial UPs were highly resistant to the commonly used AMDs such as: ampicillin, third-generation cephalosporins, ciprofloxacin, and trimethoprim-sulfamethoxazole (12–14). In Canada, Europe, and Africa, the resistance rates for ampicillin have also been found to be increasing hitting 45, 50 and 100%, respectively (5, 26–27). A range of 42.5–49.4% resistance rate towards cephalosporins has also been reported (28), whereas, Kalal and Nagaraj (20), showed 79.3% resistance for ampicillin, and 60% against cephalosporins. In the UAE, a lower resistance rate has been documented at 16.7% and 31% against expanded-spectrum cephalosporins among community and hospital-acquired UPs, respectively (29, 30).

Although trimethoprim/sulfamethoxazole has been widely used for the empirical treatment of UTI; the results of the present study showed that 40–97% and 35.6–90.2% of Gram-negative and Gram-positive isolates respectively were resistant to this drug (Tables 3–4). These figures are one of the highest reported for individual UPs. In comparison, the highest resistance rates towards this drug were 26.3% for *E. coli;* 23.3% for *Klebsiella* spp. and 16.7% for *Proteus* spp. (31). In another study from Latin America (32), the highest resistance rate was seen for *E. coli* (63%). A similar picture was seen in the case of nitrofurantoin with high resistance to this drug was observed for our Gram-negative UPs, namely: *Acinetobacter* spp. (100%); *Proteus* spp. (95%); *Pseudomonas* spp. (61%) and to a lesser extent by *Klebsiella* (43.6%) and *E. coli* (32%) (Table 3). These data are in contrast with various clinical recommendations and guidelines regarding the empirical use of trimethoprim-sulfamethoxazole and nitrofurantoin as first-line drugs of choice in the treatment of uncomplicated UTIs (32, 33).

Other widely used antibiotics for the treatment of UTIs are fluoroquinolones (34). The resistance rates among our isolates are comparable to those reported by Choe et al. (28), who also reported a very high resistance rate against fluoroquinolones among UPs isolated in a number of Asian countries with 54.9% resistance rate against ciprofloxacin, and 39% against levofloxacin. Nonetheless, our findings are much higher than those reported in several recent studies in the region and other European and North American countries (35). These high resistance levels are likely to be driven by previous exposure to fluoroquinolones, or renal transplantations which have been recently acknowledged as an independent risk factor for ciprofloxacin-resistant *E. coli* (23, 36).

Both of our *Proteus* and *Acinetobacter* spp. isolates were highly resistant to the aminoglycosides tested (gentamicin and amikacin) with 64, and 75% resistance to gentamicin respectively (Table 4). *E. coli* yielded the lowest rates of resistance towards amikacin and gentamicin with 9.5% and 18.2%, respectively which coincides with the findings of Kalal and Nagaraj (20) and Choe et al. (28). Nonetheless, these rates are once again higher than some of the published figures in other countries (32). Carbapenems (imipenem, meropenem, and ertapenem) resistance was also evident in our study with all the *Acinetobacter* spp. being resistant to ertapenem and 82–85% resistant to imipenem and meropenem too. *Proteus* and *Pseudomonas* spp. showed variable resistance patterns towards these drugs. On the contrary, *E. coli* was the least resistant UPs to the carbapenems which is in line with some other reported studies (20, 28). Other than *Klebsiella* spp., all other *Enterobacteriaceae* were susceptible to carbapenems (93%) as reported by Kalal and Nagaraj (20). In the absence of culture and sensitivity results, some consider carbapenems as an appropriate choice for the empirical treatment of UTIs (37).

As shown in Table 4, our Gram-positive isolates showed high resistance rates against the majority of the antibiotics tested. This multiple resistance spread was evident against cephalosporins, penicillin’s, aminoglycosides, erythromycin, tetracycline, clindamycin, trimethoprim-sulfamethoxazole, and imipenem. Nonetheless, 86%–96% of these isolates were susceptible to daptomycin, linezolid, vancomycin, rifampicin, and teicoplanin. These results are echoed by the findings of Bitew et al. (17) who showed that their Gram-positive UPs were highly susceptible to daptomycin, nitrofurantoin, gentamicin, vancomycin, and linezolid. The high level resistance observed against the vast majority of the commonly used antibiotics in the empirical treatment of UTIs is overwhelming. Part of this problem could be partially attributed to the irrational use of antimicrobial drugs in this locality, and the abuse of drugs by the public where patients indulge in antibiotic self-medication commonly to treat all kinds of infections has been recorded as one significant way of promoting antibiotic resistance (38). Bin Abdulhak et al. (39) reported on the non-prescription sale of antibiotics in 327 pharmacies in the capital city of Saudi Arabia and showed that 77.6% of antibiotics were dispensed without a medical prescription. Of the commonly prescribed drugs were metronidazole and ciprofloxacin being prescribed in 89% and 86% of cases with diarrhea and UTI respectively. Fortunately, a new regulation has been recently introduced in KSA to restrict the release of antibiotics without authenticated prescriptions by authorized physicians only.

In terms of individual Gram-positive bacterial isolates, the highest rates of resistance by *S. aureus* were against the penicillins. Lower resistance rates (7–15%) were recorded against trimethoprim-sulfamethoxazole, teicoplanin, vancomycin, and nitrofurantoin. However, no or low resistance rates towards daptomycin, linezolid, ciprofloxacin, or moxifloxacin by *S. aureus* was noted. In comparison, the CNS showed higher resistance patterns against the majority of the tested antimicrobial drugs. Very low resistance rates were seen against linezolid (2.4%), daptomycin (2.7%), vancomycin (6.4%) and moxifloxacin (7.7%). Of the *Streptococcus* species isolated in this study, multiple resistance patterns were also evident against cephalosporins, oxacillin, gentamicin, trimethoprim-sulfamethoxazole, clindamycin, tetracycline, and moxifloxacin. As expected the highest level of resistance patterns were recorded for *Enterococcus* species. The lowest rates of resistance were recorded for daptomycin (4.7%), and linezolid (7.5%), teicoplanin (16.7%), and 13% were designated as vancomycin resistant enterococci (VRE). In a recent study, Yang et al. (40) reported that all of their *Enterococcus* and *Staphylococcus* spp. were sensitive to linezolid, vancomycin, and teicoplanin, and suggested that fosfomycin might be an excellent treatment option for outpatients with UTIs.

Of note, the potential limitation of this study was its retrospective nature. However, the similarity of our results to those of other studies performed elsewhere confirms that such nature may unlikely affected our results. In addition, the absence of correlating AMR pattern with the clinical diagnoses cannot be considered as an important limitation of this study as this was beyond its scope and such issue need to be investigated separately.

In conclusion, this study clearly demonstrated that both Gram-positive and Gram-negative Ups in northern Saudi Arabia were highly resistant to a vast majority of the commonly used antimicrobial drugs. This indicates that it is imperative to rationalize the use of antimicrobials in hospitals and the community, and the need for countrywide campaigns for awareness to public and antimicrobial stewardship for health-care workers. Additionally, the wider and indiscriminate use of such agents by people in the community to treat these infections could also play an important role in the high resistance observed; therefore, physicians should be very careful when considering first drugs of choice for empirical treatment of UTIs in the absence of any microbiological laboratory results.
